# The Ratio of Monocytes to HDL-Cholesterol Is Associated with Cardiovascular Risk and Insulin Resistance in Patients with Rheumatoid Arthritis

**DOI:** 10.3390/life13101995

**Published:** 2023-09-29

**Authors:** Alejandro Romo-Cordero, Marta González-Sierra, Juan Carlos Quevedo-Abeledo, Adrián Quevedo-Rodríguez, Fuensanta Gómez-Bernal, Antonia de Vera-González, Raquel López-Mejías, Alejandro Jiménez-Sosa, Candelaria Martín-González, Miguel Ángel González-Gay, Iván Ferraz-Amaro

**Affiliations:** 1 Division of Internal Medicine, Hospital Universitario de Canarias, 38320 Tenerife, Spain; alexromo96co@gmail.com (A.R.-C.); mmartgon@ull.edu.es (C.M.-G.); 2 Division of Hospitalization-at-Home, Hospital Universitario de Canarias, 38320 Tenerife, Spain; martagses@gmail.com; 3 Division of Rheumatology, Hospital Universitario Dr. Negrín, 35010 Las Palmas de Gran Canaria, Spain; quevedojcarlos@yahoo.es (J.C.Q.-A.); adrian-ce@hotmail.es (A.Q.-R.); 4 Division of Central Laboratory, Hospital Universitario de Canarias, 38320 Tenerife, Spain; fuensanta95@gmail.com (F.G.-B.); adeverag@gmail.com (A.d.V.-G.); 5 Epidemiology, Genetics and Atherosclerosis Research Group on Systemic Inflammatory Diseases, Instituto de Investigación Sanitaria Marqués de Valdecilla (IDIVAL), Hospital Universitario Marqués de Valdecilla, 39011 Santander, Spain; rlopezmejias@gmail.com; 6 Research Unit, Hospital Universitario de Canarias, 38320 Tenerife, Spain; ajimenezsosa@gmail.com; 7 Internal Medicine Department, Universidad de La Laguna, 38200 Tenerife, Spain; 8 Department of Medicine and Psychiatry, Universidad de Cantabria, 39005 Santander, Spain; 9 Division of Rheumatology, IIS-Fundación Jiménez Díaz, 28040 Madrid, Spain; 10 Division of Rheumatology, Hospital Universitario de Canarias, 38320 Tenerife, Spain

**Keywords:** rheumatoid arthritis, monocyte to high-density lipoprotein cholesterol, SCORE2, insulin resistance

## Abstract

The monocytes to high-density lipoprotein (HDL)-cholesterol ratio (MHR) indicates inflammation based on the anti-inflammatory properties of HDL-cholesterol as well as the pro-inflammatory effect of monocytes. Several studies have investigated MHR in various disorders, specifically in cardiovascular diseases. Consequently, MHR has been significantly associated with cardiovascular and all-cause mortality in the general population, regardless of established risk factors. However, its role in the augmented risk of cardiovascular disease found in rheumatoid arthritis (RA) has not been studied to date. This is a cross-sectional study that encompassed 430 patients with RA and 208 controls matched by sex and age. Complete blood cell count and complete lipid profile were evaluated. Multivariable analysis was made to analyze the relationship between MHR and RA disease and features subclinical carotid atherosclerosis, and traditional CV factors including insulin resistance and beta cell function indices. MHR values did not differ between controls and patients after multivariable adjustment (12 ± 6 vs. 11 ± 6, *p* = 0.18). No relationship between this ratio and the characteristics of the disease was found excluding ESR, which showed a significant and positive association with MHR after adjustment for covariates. MHR significantly correlated with Systematic Coronary Risk Evaluation-2 (SCORE2) cardiovascular risk algorithm, and insulin resistance and beta cell function parameters after adjustment. In conclusion, MHR does not differ between patients with RA and controls. The relationship of this biomarker with disease-related data is poor. However, MHR is highly and positively related to cardiovascular risk and insulin resistance in RA.

## 1. Introduction

Rheumatoid arthritis (RA) is a chronic, systemic, autoimmune, inflammatory condition of uncertain origin that predominantly affects the synovial joints. This form of arthritis typically manifests bilaterally and, when left untreated, can result in joint damage through the erosion of cartilage and bone, ultimately leading to joint deformities. Systemic inflammation mediated by cytokines (e.g., tumor necrosis factor and interleukins) is a mean feature of the disease [1]. The central pathology of RA progresses within the synovium of diarthrodial joints, but nonarticular organs and tissues may also be affected, particularly in patients with severe joint disease. Extra-articular manifestations of RA include, among other, hematological abnormalities, the development of cardiovascular (CV) disease, dyslipidemia, and insulin resistance [2]. Common hematologic complications of RA include anemia and cytopenias that are generally associated with disease activity or severity [3]. Contrary to the general population, the dyslipidemia observed in patients with RA is paradoxical, as it is associated with inferior levels of total cholesterol and low-density lipoprotein (LDL)-cholesterol [4]. Patients with RA often experience insulin resistance and metabolic syndrome, which have been linked to the systemic inflammation of the disease [5,6,7]. Furthermore, there is strong evidence indicating that RA is a disease related to accelerated atherogenesis and increased CV morbidity and mortality [8,9].

The monocyte to HDL-cholesterol ratio (MHR) indicates inflammation based on the anti-inflammatory properties of HDL-cholesterol as well as the pro-inflammatory effect of monocytes. Several studies have investigated the relationship between MHR and prognosis in various disorders [10,11,12,13,14,15]. Most of them have focused on the relationship of this ratio with CV disease [16]. In this sense, MHR was significantly associated with all-cause and CV mortality in the general population, regardless of the established risk factors [17].

To date, there are no studies that have evaluated MHR in patients with RA. This study aimed to address this gap by investigating the relationship between MHR and RA disease characteristics, particularly focusing on subclinical atherosclerosis, dyslipidemia, and insulin resistance that accompany the disease.

## 2. Materials and Methods

### 2.1. Study Participants

We conducted a cross-sectional study that enrolled 430 individuals diagnosed with RA, recruited consecutively. Additionally, we included 208 controls matched for age and gender. Patients with RA were 18 years or older and met the 2010 ACR/EULAR classification criteria [18]. They received their diagnosis from rheumatologists and were regularly monitored in rheumatology outpatient clinics. To be eligible for this study, patients had to have a minimum RA disease duration of one year. Given that glucocorticoids are commonly prescribed for RA treatment, patients taking prednisone or an equivalent dose of 10 mg/day or less were permitted to participate. Controls were drawn from the community and recruited by general practitioners in primary care settings. However, individuals with a history of any inflammatory rheumatic disease were excluded from the control group. None of the controls were using glucocorticoids. Both patients and controls were excluded if they had a history of myocardial infarction, angina, or stroke; a glomerular filtration rate less than 60 mL/min/1.73 m^2^; a history of cancer; or any other chronic conditions such as hypothyroidism, heart or respiratory diseases, nephrotic syndrome, or evidence of an active infection. None of the patients or controls had aplasia, myeloproliferative disorders, or any other hematological diseases. The study protocol received approval from the Institutional Review Committees at Hospital Universitario de Canarias and Hospital Universitario Doctor Negrín (both in Spain), and all participants provided informed written consent (approval no. 2019-452-1). All research was conducted in compliance with applicable guidelines and regulations and in accordance with the Declaration of Helsinki.

### 2.2. Data Collection and Laboratory Assessments

Participants in the study completed a questionnaire regarding cardiovascular risk factors and medication usage and underwent a physical examination. Key metrics, including body mass index (BMI) calculated as weight in kilograms divided by the square of height in meters, abdominal circumference, and systolic and diastolic blood pressure, were measured under standardized conditions. Information on smoking status, diabetes, and hypertension was gathered from the questionnaire. Medical records were reviewed to confirm specific diagnoses and medications. Blood cell counts, including monocytes, were assessed using the Sysmex-XN automated blood cell analyzer (Sysmex, Kobe, Japan). Cholesterol, triglycerides, and HDL cholesterol levels were determined using enzymatic colorimetric assays, and LDL cholesterol was calculated using the Friedewald formula. Dyslipidemia was defined if any of the following criteria were met: total cholesterol > 200 mg/dL, triglycerides > 150 mg/dL, HDL cholesterol < 40 mg/dL in men or <50 mg/dL in women, or LDL cholesterol > 130 mg/dL. The erythrocyte sedimentation rate (ESR) and high-sensitivity C-reactive protein (CRP) were measured using standard techniques. Human interleukin 6 (IL-6) levels were determined using the electrochemiluminescence immunoassay method (Roche Diagnostics, Indianapolis, IN, USA). Serum apolipoprotein C-III was assessed using a sensitive sandwich enzyme-linked immunosorbent assay (Elabscience, Houston, TX, USA). Insulin resistance (IR) was evaluated using the homeostatic model assessment (HOMA) method, which estimated insulin sensitivity (%S) and β-cell function (%B) based on fasting plasma insulin, C-peptide, and glucose concentrations. For this study, we employed HOMA2, the updated computer-based HOMA model [19]. Disease activity in patients with RA was measured using the Disease Activity Score (DAS28) in 28 joints [20], the Clinical Disease Activity Index (CDAI) [21] and the Simple Disease Activity Index (SDAI) [22]. DAS28-ESR and DAS28-CRP were categorized according to clinical remission (<2.6), low (>2.6 to 3.2), moderate (>3.2 to 5.1), or high disease activity (>5.1) as previously described [23]. Similarly, SDAI categories were remission (<3.3), moderate (<11), high (<26), and high (>26); and CDAI was categorized in remission (<2.8), moderate (<10), high (<22), and high (>22) [24]. CV risk score SCORE2 (Systematic Coronary Risk Evaluation) was calculated according to the 2021 European Society of Cardiology Guidelines on CV disease prevention in clinical practice [25]. The SCORE2 risk categories are categorized into low- to moderate-, high-, and very high-risk groups, based on various age brackets (<50, 50–69, and ≥70 years). SCORE2 calculates an individual’s 10-year risk of experiencing fatal and non-fatal cardiovascular disease events for individuals aged 40 to 69 years. Meanwhile, for healthy individuals aged 70 years and older, the SCORE2-OP (older persons) algorithm provides estimates for 5-year and 10-year risks of fatal and non-fatal cardiovascular disease events.

### 2.3. Carotid Ultrasound Assessment

Carotid ultrasound examinations were employed to evaluate carotid intima media thickness (cIMT) in the common carotid artery and to identify focal plaques within the extracranial carotid tree among patients with RA [26]. We utilized a commercially available scanner, the Esaote Mylab 70 (Genoa, Italy), equipped with a 7–12 MHz linear transducer and an automated software-guided radiofrequency technique known as Quality Intima Media Thickness in real-time (QIMT, Esaote, Maastricht, Holland) for this purpose. In accordance with the Mannheim consensus and as previously detailed [26], the criteria for plaque presence in the accessible extracranial carotid tree (including the common carotid artery, bulb, and internal carotid artery) were defined as follows: a localized protrusion within the lumen with a minimum cIMT measurement exceeding 1.5 mm; a protrusion that was at least 50% larger than the surrounding cIMT; or an arterial lumen encroachment of more than 0.5 mm [27].

### 2.4. Statistical Analysis

Demographic and clinical characteristics of RA patients were presented as either mean with standard deviation (SD) or percentages for categorical variables. For continuous variables that did not follow a normal distribution, data were described as median with interquartile range (IQR). Univariable differences between groups were evaluated using Student’s *t*-test, the Mann–Whitney U-test, the Chi-squared test, or Fisher’s exact test, depending on the normality of the data distribution and the sample size. To analyze the association between disease-related factors and MHR, multivariable linear regression analysis was conducted, adjusting for potential confounding variables. These confounding variables were selected from demographics and traditional cardiovascular risk factors if they exhibited a *p*-value less than 0.20 in the univariate analysis with MHR. All statistical analyses were conducted with Stata software, version 17/SE (StataCorp, College Station, TX, USA), using a two-sided significance level of 5%. *p*-values below 0.05 were considered statistically significant.

## 3. Results

### 3.1. Demographic and Disease-Related Data

This study included a total of 430 patients with RA and 208 controls. Table 1 displays the demographic and disease-related characteristics of the participants. Most subjects were women (80% in both groups, *p* = 0.30), with a mean age of 56 ± 17 years in controls and 55 ± 10 years in RA patients (*p* = 0.69). Classic CV risk factors were prevalent in both groups, with no significant differences observed except for a higher prevalence of diabetes in controls. There were no significant differences in the use of statins (*p* = 0.26) and aspirin (*p* = 0.061) between controls and RA patients (Table 1).

In this cohort of RA patients, the median duration of the disease was 8 years (with an IQR of 4–15 years). At the time of the study, the mean levels of CRP and ESR were 2.7 mg/L (with an IQR of 1.3–6.1 mg/L) and 18 mm/1st hour (with an IQR of 7–32 mm/1st hour), respectively. A majority of patients, specifically 72%, tested positive for rheumatoid factor, while 65% were positive for anti-citrullinated protein antibodies. Disease activity, as assessed by DAS28-ESR, averaged 3.13 ± 1.35. Thirty-six percent of the patients were undergoing treatment with prednisone, and 87% were on at least one type of conventional disease-modifying antirheumatic drug, with methotrexate being the most commonly prescribed (73%). Additionally, 19% of patients were receiving anti-tumor necrosis factor therapies. For further details on other treatments and historical disease-related data, please refer to Table 1.

A carotid ultrasound assessment was carried out in the patients with RA. The mean carotid intima-media thickness (cIMT) was 0.696 ± 0.131 mm, and 42% of them had carotid plaques.

### 3.2. Multivariable Analysis of the Differences between Patients and Controls in Red Cells Count, Lipid Profile and Monocytes to HDL-Cholesterol Ratio

The differences in the cell blood count, lipid profile, and MHR are shown in Table 2. Several differences were found in the cell blood count. Hence, the number of red cells, hematocrit, lymphocytes, and eosinophils was significantly lower in patients with RA compared to controls. Regarding the lipid profile, the levels of total cholesterol and HDL-cholesterol were significantly higher in patients with RA compared to controls. However, although other differences in the molecules of the lipid pattern were observed, statistical significance was not reached. MHR did not differ between both groups (12 ± 6 vs. 11 ± 6, *p* = 0.18). Because this difference had a *p* value inferior to 0.20, an additional adjustment for covariates was performed (Table 2). Despite this, the MHR value difference between RA patients and controls remained non-significant.

### 3.3. Relationship of Demographics and Disease-Related Monocytes to HDL-Cholesterol Ratio in Patients with RA

Being female was significantly associated with lower MHR values compared to males. Regarding CV risk factors, the presence of obesity, diabetes, and dyslipidemia was positively and significantly related to MHR levels. Concerning disease-related data, only ESR showed a significant relationship with MHR levels after multivariable adjustment. No other disease-related information, such as disease activity or the therapies used for the management of the disease, was associated with MHR (Table 3).

### 3.4. Relationship of CV Risk Parameters to MRH in Patients with RA

MRH association with subclinical carotid atherosclerosis, SCORE2 CV risk algorithm and insulin resistance indices is shown in Table 4. A nearly significant relationship was found between the presence of carotid plaque and higher MHR levels. However, this trend did not persist after multivariable adjustment. 

The SCORE2 CV risk algorithm showed a positive and significant association with higher MHR values. This relationship was significant both when SCORE2 was considered continuous and categorical. Furthermore, after adjustment for covariates, and only in those non-diabetic patients with blood glucose lower than 110 mg/dL, a significant and positive relationship was observed between MHR values and the indices of resistance to insulin action and beta cell function (Table 4).

## 4. Discussion

To our knowledge, this is the first study in which MHR has been studied in patients with RA. According to our results, MHR does not differ between patients with RA and controls. However, MHR revealed a positive relationship with CV risk factors in RA patients and could, therefore, serve as a biomarker of CV disease risk in this population.

The number of monocytes was not different in our study between patients and controls. In contrast, HDL-cholesterol was significantly higher in patients with RA. Despite this, the MHR was not different between patients and controls after multivariable adjustment.

As mentioned above, we are not aware of previous studies on MHR in RA. However, there is some information on MHR in inflammatory or musculoskeletal diseases. In a previous study that included 323 patients with knee osteoarthritis and 283 age- and sex-matched controls, MHR was significantly higher in patients than in healthy controls [28]. Moreover, MHR significantly correlated with the radiological severity. In another study in 60 patients with psoriasis vulgaris and 50 healthy volunteers (none of the patients with psoriasis met the criteria for psoriatic arthritis), the MHR was significantly higher in patients compared to controls and was related to the area of psoriasis and the severity indices [12]. Similarly, high values of MHR have also been reported in patients with periodontal disease [29]. Contrary to those previous works, in our study, we did not find differences between patients with RA and controls. In this sense, we do not have an exact explanation for this apparent contradiction. It is possible that the modifications in the lipid pattern and blood count that occur in RA related to the disease itself differ from those that appear in other inflammatory diseases. However, since studies on MHR expression in inflammatory diseases are still limited, more information is needed to establish well-defined differences between RA and other inflammatory processes.

In our cohort of RA patients, we were unable to find significant associations between disease characteristics and MHR. Only ESR levels showed a positive and significant relationship with MHR. However, this was not the case for other acute phase reactants such as CRP or IL-6, nor for disease activity scores, extra-articular manifestations, or the use of different therapies. We have also not found a relationship between MHR and certain therapies used in the disease. This is of interest since some of them have been associated with the elevation of lipid parameters. Perhaps the cross-sectional nature of our study has prevented us from finding these associations. Another explanation could be that these treatments may modify the lipid profile but not MHR. 

MHR was positively associated with the presence of CV risk factors, such as obesity and diabetes, in the group of patients with RA. This is in line with the positive relationship of MHR with CV risk factors that has been described in the general population [30]. Furthermore, in the univariable analysis, MHR tended to be higher in patients with carotid plaque compared to those without plaque. SCORE2 includes information on non-HDL cholesterol among its variables. Given that the total cholesterol/HDL cholesterol ratio may be altered in patients with RA, the relationship between MHR and SCORE2 found in our patients with RA could be influenced by other factors related to the disease itself.

We have found a significant and independent relationship between MHR with the indices of insulin resistance and beta cell function. This fact is remarkable since insulin resistance is known to be increased in patients compared to healthy individuals. Some previous reports exist regarding the association of MHR with insulin resistance states. For instance, in a study of 605 subjects newly diagnosed with type 2 diabetes mellitus, MHR was significantly associated with higher odds of metabolic syndrome after adjustment for other confounders [31]. MHR has been also related to diabetic nephropathy and retinopathy [32]. Taking these data into account, we believe that the relationship of MHR with insulin resistance should be explored in patients with RA at a high risk of developing diabetes.

We recognize the limitation derived from the cross-sectional design of our study. For this reason, causality cannot be inferred. Furthermore, we cannot know how this marker varies throughout the progression of the disease or what the effect of different treatments is during follow-up. Radiological severity scores or accumulative steroid dosage were not collected in our study. For this reason, the relation of this information to MHR could not be assessed.

In conclusion, MHR does not differ between patients with RA and healthy controls. However, MHR denotes a relationship with classic CV risk factors in this population.

## Figures and Tables

**Table 1:** Demographics, cardiovascular risk factors, and disease-related data in RA patients.

	Controls	Rheumatoid Arthritis	
	(*n* = 208)	(*n* = 430)	*p*
Age, years	56 ± 17	55 ± 10	0.69
Female, *n* (%)	162 (79)	350 (81)	0.30
BMI, kg/m^2^	**31 ± 3**	**29 ± 15**	**0.034**
Cardiovascular risk factors and data			
Current smoker	35 (17)	93 (22)	0.16
Obesity	60 (29)	137 (32)	0.44
Hypertension	85 (41)	148 (34)	0.11
Diabetes Mellitus	**39 (19)**	**54 (13)**	**0.031**
Dyslipidemia	164 (79)	332 (77)	0.64
Statins, *n* (%)	58 (28)	139 (32)	0.26
Aspirin, *n* (%)	16 (8)	24 (10)	0.061
Carotid ultrasound			
cIMT, mm		0.696 ± 0.131	
Carotid plaque, *n* (%)		180 (42)	
Disease-related data			
Disease duration, years		8 (4–15)	
CRP at time of study, mg/L		2.7 (1.3–6.1)	
ESR at time of study, mm/1st hour		18 (7–32)	
IL-6, pg/mL		5.0 (3.2–8.6)	
Rheumatoid factor, *n* (%)		303 (72)	
ACPA, *n* (%)		253 (65)	
Swollen joints count, *n*		0 (0–1)	
Tender joints count, *n*		1 (0–4)	
DAS28-ESR		3.13 ± 1.35	
DAS28-CRP		2.73 ± 1.08	
SDAI		12 (7–19)	
CDAI		8 (4–14)	
History of extra-articular manifestations, *n* (%)		38 (10)	
Erosions, *n* (%)		166 (43)	
Current drugs, *n* (%)			
Prednisone		155 (36)	
Prednisone doses, mg/day		5 (3–5)	
NSAIDs		194 (45)	
DMARDs		373 (87)	
Methotrexate		316 (73)	
Leflunomide		94 (22)	
Hydroxychloroquine		45 (18)	
Salazopyrin		28 (7)	
Anti-TNF therapy		83 (19)	
Tocilizumab		23 (5)	
Rituximab		7 (2)	
Abatacept		12 (3)	
JAK inhibitors		20 (5)	

Data represent mean ± SD or median (IQR) when data were not normally distributed. CRP: C reactive protein; ACPA: anti-citrullinated protein antibodies. NSAID: Nonsteroidal anti-inflammatory drugs; DMARD: disease-modifying antirheumatic drug. TNF: Tumor necrosis factor; Obesity; ESR: erythrocyte sedimentation rate. JAK: Janus kinase. BMI: Body mass index; DAS28: Disease Activity Score in 28 joints. CDAI: Clinical Disease Activity Index; SDAI: Simple Disease Activity Index. cIMT: Carotid intima media thickness. Carotid ultrasound was not available for controls. Dyslipidemia was defined if one of the following was present: total cholesterol > 200 mg/dL, triglycerides > 150 mg/dL, HDL cholesterol < 40 in men or < 50 mg/dL in women, or LDL cholesterol > 130 mg/dL;. Significant *p* values are depicted in bold.

**Table 2:** Multivariate analysis of the differences between patients and controls in complete blood count, lipid profile and monocyte to HDL-cholesterol ratio.

	Controls	RA Patients			
	(*n* = 208)	(*n* = 430)	*p*	Beta Coef. (95%CI), *p*	
	Univariable			Multivariable	
Blood cells counts					
Red blood cells, × 10^6^/mm^3^	**4.71 ± 0.45**	**4.51 ± 0.40**	**<0.001**		
Hemoglobin, g/dL	13.7 ± 1.4	13.6 ± 1.3	0.099		
Hematocrit, %	**42.3 ± 3.8**	**41.5 ± 3.7**	**0.011**		
Leucocytes/mm^3^	7360 ± 1879	7158 ± 2144	0.25		
Neutrophils/mm^3^	4087 ± 1459	4056 ± 1664	0.82		
Lymphocytes/mm^3^	**2394 ± 829**	**2232 ± 837**	**0.023**		
Monocytes/mm^3^	584 ± 162	604 ± 261	0.30		
Eosinophils/mm^3^	**233 ± 173**	**183 ± 158**	**<0.001**		
Basophils/mm^3^	**40 (30–60)**	**50 (30–93)**	**<0.001**		
Platelets ×10 × 10^3^/mm^3^	264 ± 60	260 ± 64	0.44		
Lipid profile					
Total cholesterol, mg/dL	198 ± 46	206 ± 37	0.025		
Triglycerides, mg/dL	142 ± 62	153 ± 90	0.15		
HDL-cholesterol, mg/dL	**53 ± 15**	**57 ± 15**	**0.004**		
LDL-cholesterol, mg/dL	117 ± 37	119 ± 34	0.47		
LDL:HDL cholesterol ratio	2.33 ± 0.86	2.27 ± 0.96	0.48		
Non-HDL cholesterol, mg/dL	145 ± 41	150 ± 38	0.22		
Lipoprotein (a), mg/dL	41 (14–106)	33 (11–103)	0.68		
Apolipoprotein A1, mg/dL	177 ± 40	174 ± 29	0.38		
Apolipoprotein B, mg/dL	103 ± 30	108 ± 48	0.25		
Apo B:Apo A1 ratio	0.60 ± 0.18	0.63 ± 0.25	0.12		
Atherogenic index	3.9 ± 1.1	3.9 ± 1.3	0.54		
Monocytes to HDL-cholesterol ratio	12 ± 6	11 ± 6	0.18	−0.2 (−3–2)	0.86

Data represent mean ± SD or median (IQR) when data were not normally distributed. In the multivariable analysis controls is considered the reference variable. Multivariable analysis is adjusted for body mass index, smoking, hypertension, diabetes, and aspirin intake. HDL: High-density lipoprotein; LDL: low-density lipoprotein. Significant *p* values are depicted in bold.

**Table 3:** Relationship of demographics and disease related to monocytes to HDL-cholesterol ratio in RA patients.

	Monocytes to HDL Cholesterol Ratio			
	Beta Coefficient (95%CI), *p*			
	Univariable		Multivariable	
Age, years	0.05 (−0.02–0.1)	0.15		
Female, *n* (%)	**−4 (−5–2−2)**	**<0.001**		
BMI, kg/m^2^	0.02 (−0.02–0.06))	0.30		
Cardiovascular risk factors				
Current smoker	1 (−0.2–3)	0.099		
Obesity	**2 (0.3–3)**	**0.020**		
Hypertension	1 (−0.2–2)	0.093		
Diabetes Mellitus	**2 (0.2–4)**	**0.030**		
Dyslipidemia	**2 (0.4–3)**	**0.013**		
Statins, *n* (%)	0.5 (−0.8–2)	0.44		
Aspirin, *n* (%)	1 (−2–4)	0.46		
Disease-related data				
Disease duration, years	−0.03 (−0.1–0.04)	0.37		
CRP at time of study, mg/L	0.04 (−0.01–0.08)	0.15	0.02 (−0.02–0.08)	0.34
ESR at time of study, mm/1st hour	**0.03 (0.007–0.06)**	**0.013**	**0.03 (0.007–0.06)**	**0.012**
IL-6, pg/mL	0.00006 (−0.04–0.04)	0.99		
Rheumatoid factor, *n* (%)	−0.4 (−2–1)	0.59		
ACPA, *n* (%)	−0.4 (−2–1)	0.60		
Swollen joints count, *n*	−0.1 (−0.5–0.3)	0.58		
Tender joints count, *n*	−0.1 (−0.3–0.06)	0.19	−0.1 (−0.3–0.06)	0.19
DAS28-ESR	−0.09 (−0.5–0.4)	0.70		
DAS28-CRP	0.07 (−0.5–0.7)	0.81		
SDAI	0.02 (−0.02–0.06)	0.33		
CDAI	−0.02 (−0.1–0.06)	0.66		
History of extraarticular manifestations, *n* (%)	0.9 (−1–3)	0.36		
Erosions, *n* (%)	−0.2 (−2–1)	0.79		
Current drugs, *n* (%)				
Prednisone	−0.3 (−2–1)	0.62		
Prednisone doses, mg/day	−0.1 (−0.4–0.2)	0.42		
NSAIDs	−0.8 (−2–0.4)	0.19	−0.3 (−2–1)	0.65
DMARDs	0.6 (−1–2)	0.53		
Methotrexate	−0.3 (−2–1)	0.65		
Leflunomide	0.5 (−1–2)	0.55		
Hydroxychloroquine	−0.4 (−3–2)	0.71		
Salazopyrin	−0.7 (−3–3)	0.97		
Anti-TNF therapy	0.6 (−1–2)	0.50		
Tocilizumab	−0.5 (−3–2)	0.70		
Rituximab	−1 (−6–4)	0.59		
Abatacept	−0.04 (−4–4)	0.99		
JAK inhibitors	−0.7 (−4–2)	0.67		

Monocytes to HDL cholesterol is considered the dependent variable in this analysis. NSAID: Nonsteroidal anti-inflammatory drugs; DMARD: disease-modifying antirheumatic drug. TNF: tumor necrosis factor; Obesity; ESR: erythrocyte sedimentation rate; JAK: Janus kinase. BMI: Body mass index; DAS28: Disease Activity Score in 28 joints. CRP: C reactive protein. ACPA: Anti-citrullinated protein antibodies; HOMA: homeostatic model assessment. CDAI: Clinical Disease Activity Index; SDAI: Simple Disease Activity Index. IL-6: interleukin 6. Dyslipidemia was defined if one of the following was present: total cholesterol > 200 mg/dL, triglycerides > 150 mg/dL, HDL cholesterol < 40 in men or < 50 mg/dL in women, or LDL cholesterol > 130 mg/dL. Significant *p* values are depicted in bold. Multivariable analysis is adjusted for age, sex, smoking, obesity, diabetes, and dyslipidemia.

**Table 4:** Relationship of cardiovascular risk parameters to monocytes to HDL-cholesterol ratio in RA patients.

		Monocytes to HDL Cholesterol Ratio			
		Beta Coefficient (95%CI), *p*			
		Univariable		Multivariable	
Carotid ultrasound					
cIMT, mm	0.0696 ± 0.131	2 (−3–7)	0.43		
Carotid plaque, *n* (%)	180 (42)	1 (−0.1–2)	0.074	0.5 (−0.9–2)	0.48
SCORE2					
SCORE2, %	**3.6 (1.8–5.8)**	**0.4 (0.2–0.5)**	**<0.001**		
Low or moderate risk	282 (66)	ref.			
High risk	**112 (26)**	**1.6 (0.2–3.0)**	**0.021**		
Very high risk	**36 (8)**	**5.3 (2.8–7.8)**	**<0.001**		
Insulin resistance indices *					
Glucose, mg/dL	87 ± 10	0.02 (−0.05–0.08)	0.56	0.01 (−0.05–0.0006)	0.68
Insulin, µU/mL	**7.7 (5.2–12.4)**	**0.1 (0.08–0.2)**	**<0.001**	**0.1 (0.06–0.2)**	**<0.001**
C-peptide, ng/mL	**2.32 (1.51–3.44)**	**0.7 (0.04–1)**	**<0.001**	**0.6 (0.3–0.9)**	**<0.001**
HOMA2-IR	**1.00 (0.66–1.56)**	**1 (0.6–2)**	**<0.001**	**1 (0.5–2)**	**0.001**
HOMA2-S%	**118 ± 76**	**−0.02 (−0.02–(−0.009))**	**<0.001**	**−0.01 (−0.02–(−0.005))**	**0.001**
HOMA2-B%-C-peptide	**165 ± 75**	**0.02 (0.01–0.03)**	**<0.001**	**0.02 (0.009–0.03)**	**<0.001**

Data represent means ± SD or median (IQR) when data were not normally distributed. Monocytes to HDL cholesterol ratio is considered the dependent variable in this analysis. SCORE: Systematic Coronary Risk Evaluation. * Insulin resistance analysis is only performed for non-diabetic patients and if glucose is lower than 110 mg/dL (*n* = 339). cIMT: carotid intima media thickness. HOMA: homeostatic model assessment, CI: confidence interval. Multivariable analysis is adjusted for age, sex, smoking, obesity, diabetes, and dyslipidemia. SCORE2 calculator relation to Monocytes to HDL cholesterol is not adjusted. Significant *p* values are depicted in bold.

## Data Availability

The data sets used and/or analyzed in the present study are available from the corresponding author upon request.

## Abbreviation

ACPA: Anti-citrullinated protein antibodies, BMI: body mass index, CDAI: Clinical Disease Activity Index, CI: Confidence interval, cIMT: carotid intima media thickness, CRP: C reactive protein, CV: Cardiovascular, DAS28: Disease Activity Score in 28 joints, DMARD: disease-modifying antirheumatic drug, ESR: erythrocyte sedimentation rate, HDL: High density lipoprotein, HOMA: Homeostatic model assessment, IQR: interquartile range, LDL: Low density lipoprotein MHR: monocyte to HDL-cholesterol ratio, NSAID: Nonsteroidal anti-inflammatory drugs, TNF: tumor necrosis factor, SCORE: Systematic Coronary Risk Evaluation, SCORE-OP: Systematic Coronary Risk Evaluation Older person, JAK: Janus kinase, RA: Rheumatoid arthritis, SD: Standard deviation, SDAI: Simple Disease Activity Index.
